# Contextual factors influencing incomplete immunization and investigation of its geospatial heterogeneity in Pakistan: a cross-sectional study based on PDHS (2017–18)

**DOI:** 10.1186/s12889-023-16508-8

**Published:** 2023-08-24

**Authors:** Asifa Kamal, Ayesha Waseem, Maryam Siddiqa, Muhammad Ijaz, Abeera Shakeel, Soofia Iftikhar

**Affiliations:** 1 Department of Statistics, Lahore College for Women, University, Lahore, Pakistan; 2 Department of Mathematics and Statistics, International Islamic University Islamabad, Islamabad, Pakistan; 3 Department of Mathematics and Statistics, The University of Haripur, Haripur, Pakistan; 4 Department of Statistics, Shaheed Benazir Bhutto Women University Peshawar, Peshawar, Pakistan

**Keywords:** Incomplete immunization, Spatial Analysis, Hotspot Analysis, GWR4, PDHS (2017–18)

## Abstract

**Background:**

Immunization is one of the most effective public health initiatives, saving millions of lives and lowering the risk of diseases such as diphtheria, tetanus, influenza, and measles. Immunization saves an estimated 2–3 million lives per year. A study of the regional variations in incomplete immunization will be useful in identifying gaps in the performance of immunization programs that are not noticed by standard vaccination programs monitoring. The primary goal of this study was to identify factors influencing child immunization status and to examine regional variations in incomplete immunization among children aged 12 to 23 months in Pakistan.

**Methods:**

For the current study, the data were taken from the Demographic and Health Survey for Pakistan (PDHS 2017–2018). Ever-married women who had children aged 12–23 months were included in this study. The immunization status of children was used as an outcome variable. In order to determine the effects of different factors on incomplete immunization, multilevel logistic model was used. To study the geographical variation of incomplete immunization, hotspot analysis was done using ArcGIS 10.7 and SaTScan software and to identify significant predictors of incomplete immunization, GWR 4 software was used.

**Results:**

Place of delivery, gender of child, mother’s educational level and region were identified as significant determinants of incomplete immunization of children in Pakistan. Chances of incomplete immunization of children were found significantly lower for educated mothers (AOR = 0.52, 95% CI 0.34–0.79) and mothers who had delivered children in the health facilities (AOR = 0.51, 95% CI 0.32–0.83). Female children were more likely (AOR = 1.44, 1.95% CI 1.04–1.99) to be incompletely immunized as compared to male children. FATA (AOR = 11.19, 95% CI 4.89–25.6), and Balochistan (AOR = 10.94, 95% CI 5.08–23.58) were found at the highest risk of incomplete immunization of children as compared to Punjab.

The significant spatial heterogeneity of incomplete immunization was found across Pakistan. The spatial distribution of incomplete immunization was clustered all over Pakistan. The high prevalence of incomplete immunization was observed in Balochistan, South Sindh, North Sindh, South KPK, South FATA, Gilgit Baltistan, Azad Jammu Kashmir, South and East Punjab. Drang and Harcho were identified as hotspot areas of incomplete immunization in Gilgit Baltistan. Secondary clusters with a high risk of incomplete immunization were found in regions Balochistan, Sindh and FATA.

**Conclusion:**

Gender biasedness towards female children, regarding complete immunization of children prevailed in Pakistan. Spatial heterogeneity was also found for incomplete immunization of children. To overcome the problem access to health facilities is the foremost step. Government should target hotspot areas of incomplete immunization of children to provide primary health care facilities by opening health care units in these areas. The government in collaboration with the media should launch awareness campaigns in those areas to convince people that complete immunization is the right of every child regardless of gender.

## Background

Child immunization is one of the most effective public health interventions as it protects a child against vaccine preventable diseases like tuberculosis, pertussis, diphtheria, tetanus, influenza, measles and polio [1]. Every year immunization saves 2–3 million lives from death [2]. Child immunization is also one of the most cost-effective steps to reduce the risk of morbidity and mortality from vaccine prevalent disease [3, 4].

World Health Organization (WHO) launched Expanded Program on Immunization (EPI) in 1974, with the purpose of vaccinating each child in the world. In Pakistan, the Expanded Program on Immunization (EPI) was started in 1978 with the purpose of expanding the movement about vaccination. The Global Alliance for Vaccines and Immunization (GAVI) is also one of the efforts to ensure the complete immunization of children and strengthen the availability of immunization services [5]. In South Asia, Pakistan was the first country who had introduced new vaccines and added in the immunization schedule with the help of GAVI [6].

Under five child mortality has been taken as the most important measure to assess the socioeconomic development of a country [7]. Globally 6.3 million children died under five years of age due to vaccine preventable diseases [8] and around 23 million children in the world remain unvaccinated [2]. It was also reported in the literature that one out of every five children is deprived of vaccination [9]. Another study estimated that 1.5 million children under the age of five years remain unvaccinated [10]. In 2018, the global percentage of under vaccinated and non-vaccinated children were reported 49.5% and 19.2% respectively [11]. The majority of unvaccinated children were from Asian and African countries [12]. In 2007, 27 million infants did not receive measles vaccination [13]. Almost 1,500,000 children globally, died from vaccine preventable diseases in 2013 [14].

In 2013, 192 deaths from measles were reported in Punjab and 83 cases of polio were reported in Pakistan [1]. In PDHS 2012–13, it was reported that only 54% of children between 12–23 months of age were fully vaccinated which was quite low than the target of 90% vaccinated children till 2020 [15]. The proportion of incomplete immunization in Pakistan varies from 37–58% [1]. Geographical clustering of children who are at high risk of vaccine preventable disease was reported using spatial analysis techniques, in various studies [16–18]. In this study, spatial analysis is also performed to investigate the prevalence of incomplete immunization at each cluster level and to identify the areas where children are at increased risk of vaccine preventable disease in Pakistan.

Effective strategies for the vaccine preventable disease control program can be formulated by accounting for geographical variation in incomplete immunization that lead toward evidence-based decision making. A study of spatial variation of incomplete immunization could be also helpful to identify the gap in immunization program performance that is not detected by the usual monitoring of vaccination programs.

In this study, the primary objective is to determine factors affecting child immunization status, and secondly, to observe spatial variations of incomplete immunization in Pakistan among children 12–23 months of age.

## Methods

### Data, study design and study setting

Pakistan is located in North-Western part of South-Asia having latitude and longitude (30.3753^o^ N, 69.3451^o^ E), covering 881,912 km^2^ and ranked 33^rd^ largest country by area [19]. About 297,635 square miles area of Pakistan are covered by land and 9,737 square miles are covered by water [19]. Pakistan is a country with six regions named as Punjab, Sindh, Khyber Pakhtunkhwa, Balochistan, Azad Jammu Kashmir, and Gilgit Baltistan and two territory areas Islamabad Capital Territory (ICT) and Federal Administered Tribal Areas (FATA).

For the current study, the data were taken from the nationally representative authentic, Pakistan Demographic and Health Survey (PDHS, 2017–18) conducted by the National Institute of Population Studies (NIPS) with the assistance and technical aid of ICF, United Nations Population Fund (UNFPA), United States Agency for International Development USAID and Department for International Development (DFID) [20]. Spatial data has been accessed from ICF [21]. Children immunization data has been downloaded from the DHS measure website after permission from the relevant authority [22].

### Inclusion/exclusion criteria

#### Inclusion criterion

Following the guidelines of DHS [23], cohort of living children aged 12–23 months, born to mothers who were aged 15–49 years at the time of survey were included in the study.

#### Exclusion criterion

Children below age 12 months and children not alive at the time of survey were excluded. Children aged 12–23 months were selected as the children of this age group had completed their vaccination schedule. Cases with missing observations were also excluded.

Two stage stratified random sampling design was used in PDHS 2017–18. Women were nested in households and households were nested in enumeration blocks (EBs) in that survey. Data about children were collected from ever-married women of reproductive age (15–49 years). The sample comprised 12,708 children under five years of age but in the current study only children of 12–23 months of age were selected. After imposing that restriction sample was reduced to 2484 children of 12–23 months of age.

### Outcome variable

In this study, the outcome variable is child immunization status, categorized as completely immunized and incompletely immunized. In this study completely immunized was coded as ‘0’ and incompletely immunized was coded as ‘1’.

### Operational definition

#### Completely immunized

A child was considered completely immunized if he/she had received one dose of BCG, three doses of polio and DPT, and one dose of measles.

### Incompletely immunized

A child was considered incompletely immunized if he/she had missed at least one dose of vaccines.

### Independent variables

The individual and community level characteristics of women were selected from relevant literature as the potential factors of incomplete immunization. The variables of individual level characteristics were the place of delivery, gender of the child, mother’s occupation, father’s educational level, antenatal visits, gender of household head, media exposure, mother’s educational level, age of mother, and birth order. The community level variables included were region and place of residence.

### Data extraction and management

Children immunization data and other variables relevant to the study were extracted from the child recode file (PKKR71.DTA). In geographical coordinate file, cluster data were used from 561 clusters or enumeration blocks (EBs). In the geographic coordinate data file 535^th^ enumeration block was recorded as 0, so this EB was dropped from the study. After getting data, data extraction, data weighting, and data recording were carried out using STATA V.15 while for spatial analysis data were managed using Excel and STATA V.15. Following is the sampling procedure layout.

### Statistical analysis

#### Multilevel logistic regression model

The PDHS 2017–18 data was of hierarchical nature as the children born to women (level 1) were nested within households (level 2). Further, households were nested within clusters/Enumeration Blocks (EB’s) at level 3. Multilevel is appropriate choice when data has been drawn from different levels (level 2 and 3 in current study i.e. household and clusters/EB’s respectively) and unit of analysis is measured at the lowest level i.e. children. The dependent variable i.e. incomplete immunization of the child was binary, so a multilevel binary logistic regression model was selected to determine the factors affecting incomplete immunization.

Four models were fitted in the current study. The null model was run to check the variation in incomplete immunization due to clusters. The null model in multilevel model is used to check whether or not the multilevel model is required to fit the data. It is the baseline model where the dependent variable is not conditioned by any independent variables. In null model, random effect is computed along with fixed effects to find whether intercepts vary between clusters. That is why it is also called “random intercept only model”. An unadjusted model was fitted to check the effect of each independent variable without adjusting the effect of other independent variables and odd ratios produced were reported as Unadjusted Odd Ratio (UOR).

In Model I, all individual level factors were used to compute the Adjusted Odd Ratio (AOR) in order to determine the effect of these factors on incomplete immunization. The Model II was fitted using only community level characteristics of women. In Model III, all individual and community level characteristics of women were combined.

### Spatial statistical analysis

#### Spatial autocorrelation analysis

Spatial autocorrelation analysis was performed to identify the distribution of incomplete immunization in Pakistan whether it is dispersed, clustered, or randomly distributed. Spatial autocorrelation was tested under the null hypothesis that incomplete immunization is randomly distributed all over Pakistan. It computes Moran’s I value to identify the distribution. If Moran’s I value is -1 then the distribution is dispersed, if it is + 1 then the distribution is clustered, and if it is 0 then incomplete immunization is distributed randomly.

### Hotspot analysis

The hotspot analysis detects areas with a high prevalence of incomplete immunization across Pakistan. It identifies the location of statistically significant hot spot and cold spot areas of incomplete immunization. The hotspot analysis includes.

- Local Moran’s I analysis
- Geits Ord-Gi* analysis
- Spatial scan statistical analysis

### Local Moran’s I analysis

Local Moran’s I analysis detects clusters and outliers of incomplete immunization. It identifies positively correlated HH (high high) and LL (low low) clusters and negatively correlated HL (high low) and LH (low high) outliers. The same proportion of incomplete immunization all over the area, either high or low, considered as cluster while the area with a high proportion of incomplete immunization surrounded by a low proportion of incomplete immunization is considered an outlier. The positive value of Moran’s I is identified as a cluster and the negative value is identified as an outlier.

### Getis Ord-Gi* analysis

The Getis Ord-Gi* analysis detects statistically significant clusters with a prevalence of incomplete immunization. The Getis Ord-Gi* analysis identify the clusters with high and low proportion for incomplete immunization.

### Spatial scan statistical analysis

Spatial scan statistical analysis further strengthens the findings of hotspot analysis by determining the geographical location of significant clusters identified as hotspots. The spatial scan statistical analysis was performed using Kuldoff’s SaTScan version 9.6 software. The circular scanning window was used to identify the significant clusters of incomplete immunization. A child who was completely immunized was treated as a control and a child who was incompletely immunized was treated as a case. The spatial scan statistical analysis identifies primary and secondary clusters with a high prevalence of incomplete immunization by computing the relative risk and likelihood ratio for incomplete immunization.

### Spatial interpolation

Interpolation is a technique to determine the proportion of incomplete immunization in un-sampled areas on the bases of sampled areas. In geostatistical spatial interpolation, different interpolation techniques are included as ordinary kriging, simple ordinary and empirical Bayesian kriging [24]. After comparing the root mean square error, the ordinary kriging method was utilized for developing spatially distributed map of un-sampled areas with incomplete immunization over the study area for PDHS (2017–18).

### Geographically Weighted Regression (GWR)

In order to identify the predictors of observed spatial patterns of incomplete immunization geographically weighted regression was used [25]. Global geographical weighted regression along with local geographical weighted regression was performed. The variables with 60% and above significance were selected for geographically weighted regression [13]. The spatial behavior of predictors all over Pakistan was represented graphically using ArcGIS software.

## Results

From Table 1, it can be seen that a large number of children remain unvaccinated against DPT3 and Measles 1^st^ dose about 24.6% and 27.2%, respectively while only 65.7% of children were completely immunized in PDHS (2017–18).

**Table 1:** Percentage distribution of immunization among children 12–23 months of age, PDHS (2017–2018)

Vaccine	PDHS (2017–18)	
	**Vaccinated**	**Unvaccinated**
**BCG**	87.8	12.2
**DPT 1**	86.3	13.7
**DPT 2**	82.5	17.5
**DPT 3**	75.4	24.6
**Polio 1**	94.8	5.2
**Polio 2**	90	10
**Polio 3**	86.2	13.8
**Measles 1**	72.8	27.2
**All Vaccines**	65.7	34.3

### Individual and community level characteristics of respondents

Table 2 shows that out of 2484 children, 71.8% of children were born in health facilities, and 51.9% of them were male. About 82.7% of children’s mothers were not working and 29.2% of children were born to uneducated fathers. The majority of mothers followed more than four antenatal health care visits. Only 11% of households had a female head of household and 37.4% of mothers in Pakistan were deprived of access to media. Around 53.4% of children had educated mothers and 57.9% of mothers were 25-34 years of age and 64.3% of children had birth order 1-3.

**Table 2:** Percentage distribution of children aged 12–23 months across individual & community level factors affecting incomplete immunization among, PDHS (2017–18)

Individual & Community Level Characteristics	Unweighted Frequency		Weighted Frequency	
	**N**	**%**	**N**	**%**
**Place of Delivery**				
Home	726	29.2	725	28.1
Health Facility	1758	70.8	1757	71.8
**Gender of Child**				
Male	1272	51.2	1272	51.9
Female	1212	48.8	1212	48
**Mother’s Occupation**				
Not Working	2170	87.3	2170	82.7
Working	314	12.7	314	17.3
**Father’s Educational Level**				
Not Educated	646	26.3	646	29.2
Educated	1812	73.7	1812	70.8
**Antenatal Visits**				
Less than 4 Visits	1091	49.7	1091	47.6
4 or more Visits	1102	50.3	1102	52.4
**Gender of Household Head**				
Male	2232	89.9	2232	89
Female	252	10.1	252	11
**Media Exposure**				
No	955	38.5	955	37.4
Yes	1526	61.5	1526	62.5
**Mother Education**				
Not Educated	1186	47.8	1186	46.5
Educated	1298	52.2	1298	53.4
**Age of Mother**				
15—24 years	685	27.6	685	28.7
25—34 years	1414	56.9	1414	57.9
35—49 years	385	15.5	385	13.3
**Birth Order**				
1—3	1596	64.3	1596	64.3
4—6	666	26.8	666	27.6
> = 7	222	8.9	222	8.1
**Region**				
Punjab	572	23.0	572	54.2
Sindh	419	16.9	419	22.0
KPK	421	16.9	421	16.3
Baluchistan	261	10.5	261	4.4
ICT	166	6.7	166	0.8
FATA	193	7.8	193	2.2
Gilgit Baltistan	168	6.8	––-	––-
AJK	284	11.4	––-	––-
**Type of Place of Residence**				
Urban	1110	44.7	1110	34.3
Rural	1374	55.3	1374	65.7

In the PDHS (2017–18), about 54.2% of children resided in the region Punjab, 22.0% in Sindh, while 16.3% lived in KPK. The percentage of children in the study who lived in rural areas was almost 66%, while 34% of children were from urban areas.

### Multilevel binary logistic model

In order to identify the significant factors affecting incomplete immunization, a multilevel binary logistic model was used. Four different models were fitted, null model, individual-level factors model (Model I), community-level factors model (Model II), and combined model of individual and community-level factors (Model III). The place of delivery, gender of child, gender of household head, mother’s education and media exposure were identified as significant factors of Model I (Individual level factors model). Region, and place of residence were found as the significant factors of incomplete immunization with *p*-value < 0.05 for Model II (Community level factors model).

The results of Table 3 showed that, in individual level factor model, the child born in the health facility had 48% fewer chances to become incompletely immunized (AOR = 0.52, 95% CI 0.32–0.84). It was observed that female children had more risk of incomplete immunization (AOR = 1.44, 95% CI 1.04–1.99) than male children in Model III. In Model I, it was found that child belonged to a household headed by a woman had significantly 53% less risk of being incompletely immunized (AOR = 0.47, 95% CI 0.24–0.94) and in Model III, child had 47% less chance of incomplete immunization if he lived under a supervision of woman headed household (AOR = 0.53, 95% CI 0.27–1.05). The child of educated women had a 48% less chance of being incompletely immunized as compared to those who were born to uneducated mothers (AOR = 0.52, 95% CI 0.34–0.79) in Model III.

**Table 3:** Multilevel binary logistic regression model identifying factors affecting incomplete immunization in Pakistan, PDHS (2017–18)

Individual & Community Level Characteristics	Null Model	Bivariate Analysis		Model I		Model II		Model III	
		**UOR**	**95% CI**	**AOR**	**95% CI**	**AOR**	**95% CI**	**AOR**	**95% CI**
**Place of Delivery (ref. = Home)**									
Health Facility	––-	0.41	(0.28–0.62)*	0.52	(0.32–0.84)*	––	––	0.51	(0.32–0.83)*
**Gender of Child (ref. = Male)**									
Female	––-	1.43	(1.04—1.97)*	1.45	(1.05–2.01)*	––	––	1.44	( 1.04–1.99)*
**Mother Occupation (ref. = Not working)**									
Working	––-	1.04	(0.58—1.87)	0.80	(0.44–1.48)	––	––	0.92	(0.50–1.70)
**Father Educational Level (ref. = Not educated)**									
Educated	––-	0.44	(0.29 – 0.68)*	0.70	(0.45–1.09)	––	––	0.69	(0.43–1.08)
**Antenatal Visits (ref. = Less than 4 visits)**									
4 or more visits	––-	0.44	(0.30–0.64)*	0.78	(0.52–1.17)	––	––	0.80	(0.54–1.20)
**Gender of Household Head(ref. = Male)**									
Female	––-	0.53	(0.28—1.01)*	0.47	(0.24–0.94)*	––	––	0.53	(0.27–1.05)
**Media Exposure (ref. = No)**									
Yes	––-	0.39	(0.25—0.60)*	0.59	(0.38–0.90)*	––	––	0.65	(0.42–1.01)
**Mother Educational Level (ref. = Not educated)**									
Educated	––-	0.30	( 0.20—0.46)*	0.42	(0.28–0.64)*	––	––	0.52	(0.34–0.79)*
**Mother Age (ref. = 15–24)**									
25–34	––-	1.08	(0.78—1.50)	1.10	(0.73–1.66)	––	––	1.18	(0.77–1.79)
35–49	––-	1.28	(0.75—2.20)	0.90	(0.44–1.84)	––	––	0.95	(0.46–1.96)
**Birth Order (ref. = 1–3)**									
4–6	––-	1.16	(0.77–1.74)	0.97	(0.58–1.60)	––-	––-	0.94	(0.57–1.57)
> = 7	––-	2.51	(1.43–4.41)*	1.60	(0.77–3.31)	––-	––-	1.40	(0.67–2.93)
**Region (ref. = Punjab)**									
Sindh	––-	5.22	(2.99—9.14)	––	––-	5.77	(3.28–10.16)*	4.88	(2.75–8.63)*
KPK	––-	4.30	(2.25—8.22)	––	––-	4.06	(2.13–7.72)*	3.41	(1.85–6.29)*
Balochistan	––-	21.81	(10.44 -45.57)	––	––-	22.02	(10.58–45.85)*	10.94	(5.08–23.58)*
ICT	––-	2.82	(1.48—5.39)	––	––-	3.04	(1.50–6.14)*	4.12	(2.15–7.92)*
FATA	––-	24.30	(10.60 -55.69)	––	––-	19.30	(8.39–44.37)*	11.19	(4.89–25.6)*
**Type of Place of Residence (ref. = Urban)**									
Rural	––-	2.14	(1.34—3.41)	––	––-	1.98	(1.27–3.07)*	1.08	(0.66–1.75)
** Random Effects**	3.18	––-		2.27		2.18		1.80	
** Random Intercept Variance**	0.05	––-		0.68		0.03		0.27	
** AIC**	2123.92	––-		1765.75		2038.35		1717.40	
** BIC**	2134.99	––-		1841.52		2082.64		1825.64	
** ICC**	0.49	––-		0.41		0.39		0.35	

^*^significant at 5%

In the community characteristics model (Model II), the children resided in FATA had 19.3 times more risk of incomplete immunization (AOR = 19.3, 95% CI 8.39–44.37) while children who belonged to Balochistan had 22.02 times higher risk of incomplete immunization (AOR = 22.02, 95% CI 10.58–45.85) as compared to the children who lived in Punjab. In Model III when community level and individual factors were combined in the model, chances of incomplete immunization reduced as compared to Model I, but the effect remained significant. Children who resided in FATA were more likely to become incompletely immunized as compared to the children who lived in Punjab (AOR = 11.2, 95% CI 4.89–25.6). The risk of incomplete immunization is found almost twice in children who resided in rural areas as compared to urban children (AOR = 1.98, 95% CI 1.27–3.07) in only community level factor model but when controlled for individual level factors, the effect is reduced and also turned insignificant (AOR = 1.08, 95% CI 0.66–1.75).

The ICC value is computed for each model, the ICC value in the null model is 0.49, indicating that, due to variability between clusters, and there is a 49% variation in incomplete immunization. For Model I, it was 0.41 and for Model III, the ICC value was the lowest at 0.35, indicating variability in clusters. Model III is determined as the best model with the lowest AIC and BIC values. The final model (Model III) identified the place of delivery, gender of child, mother’s educational level and region as significant determinants of incomplete immunization of children in Pakistan.

### Global spatial autocorrelation (Global Moran’s I)

**Fig. 1:**
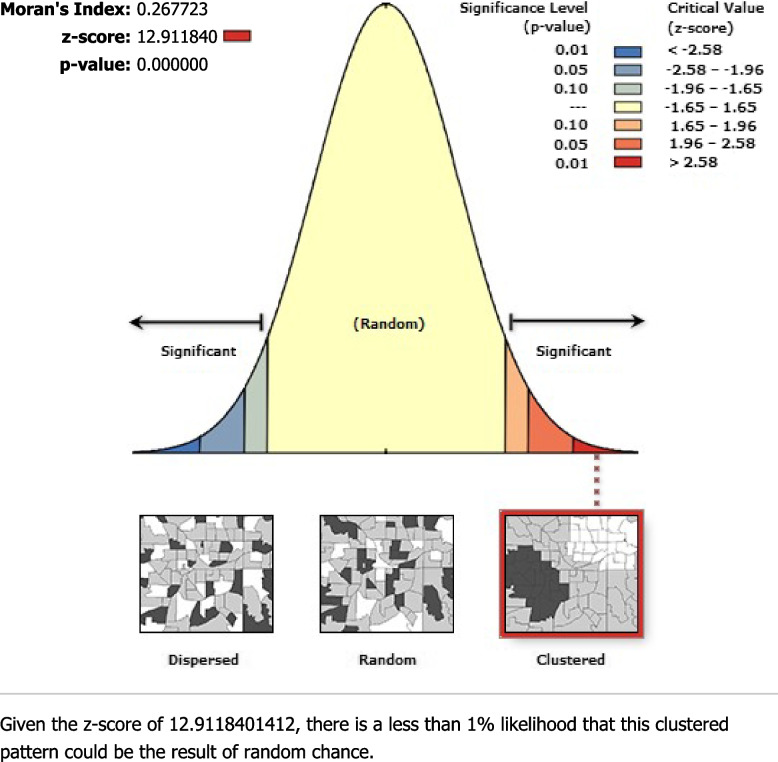
Global Spatial Autocorrelation of Incomplete Immunization in Pakistan

The Global spatial autocorrelation identifies, the spatial distribution of incomplete immunization that was clustered all over Pakistan (Fig. 1). The significant small p-value and large z-score reject the null hypothesis that the distribution of incomplete immunization is randomly distributed.Moran’s I p-value showed the presence of significant variation in incomplete immunization across Pakistan. The positive Moran’s Index value (0.267723) showed a tendency to move toward clustering. The statistically significant Moran’s I (*p*-value < 0.05) lead to confirm the existence of spatial autocorrelation.

### Cluster and outlier (Local Moran’s I) analysis

Local Moran’s I analysis detected clusters and outliers of incomplete immunization in Pakistan. It revealed that there are significant clusters and outliers in the study area. The high-low outliers are the clusters with a high proportion of incomplete immunization surrounded by a low proportion of incomplete immunization while low–high outliers are the clusters with a low proportion of incomplete immunization surrounded by a high proportion of incomplete immunization.

**Fig. 2:**
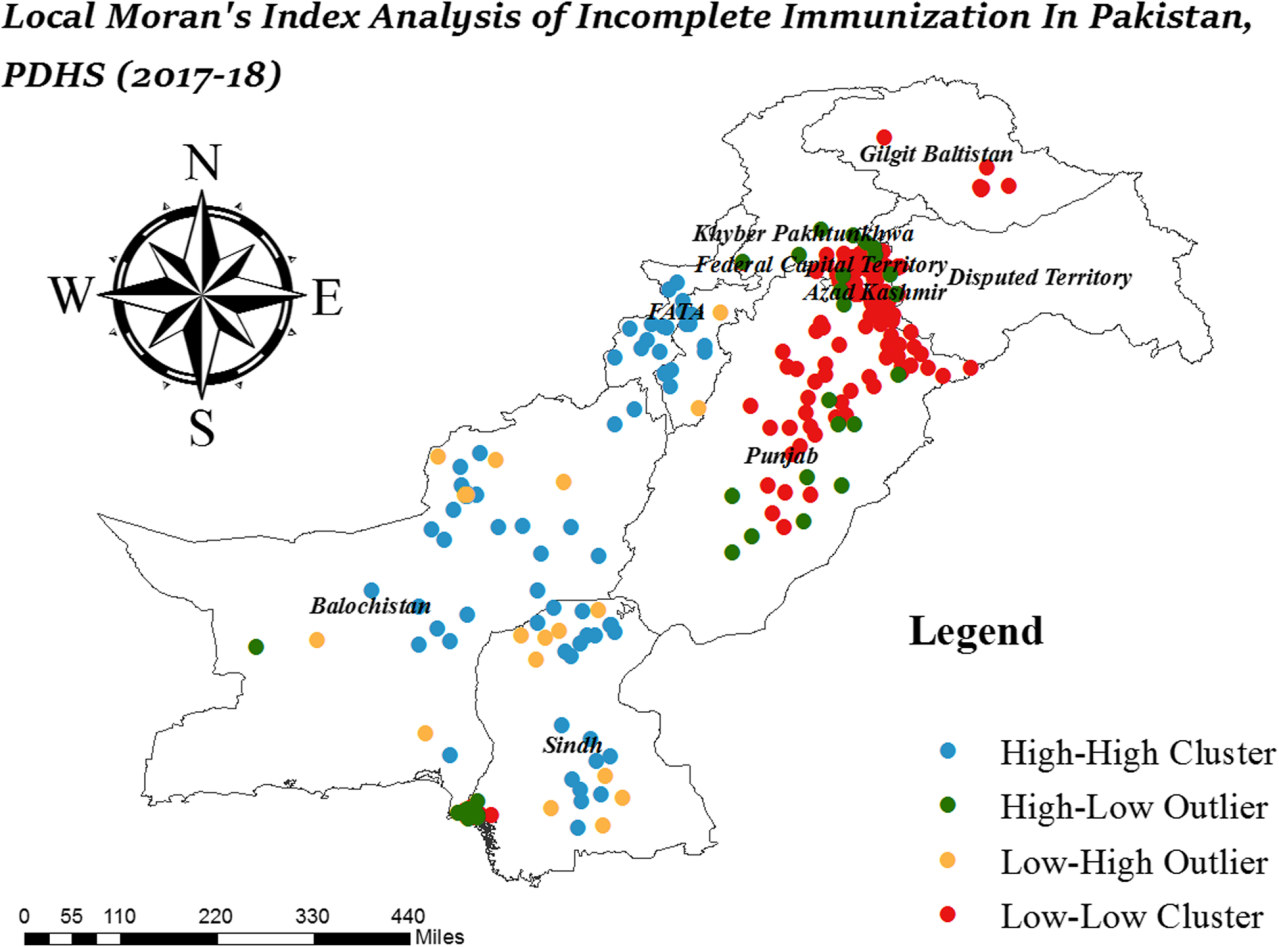
Cluster and Outlier Analysis of Incomplete Immunization in Pakistan; Shape file source: www.giszoo.com, Map output: own analysis on ArcGIS

Figure 2 shows the clusters with a high proportion of incomplete immunization observed in Sindh, Balochistan, South-West FATA and South-West KPK. Punjab, Federal Capital Territory, Azad Jammu Kashmir, and Gilgit Baltistan were observed with a low proportion of incomplete immunization. The high-low outliers were observed in Punjab, Federal Capital Territory, South Sindh, Azad Jammu Kashmir, West Balochistan and South-East KPK while low–high outliers were observed in Sindh, Balochistan, and East KPK. Overall, Balochistan, Sindh, KPK, and FATA were the regions where the proportion of incomplete immunization was high in Pakistan.

### Getis-Ord-Gi* analysis

**Fig3:**
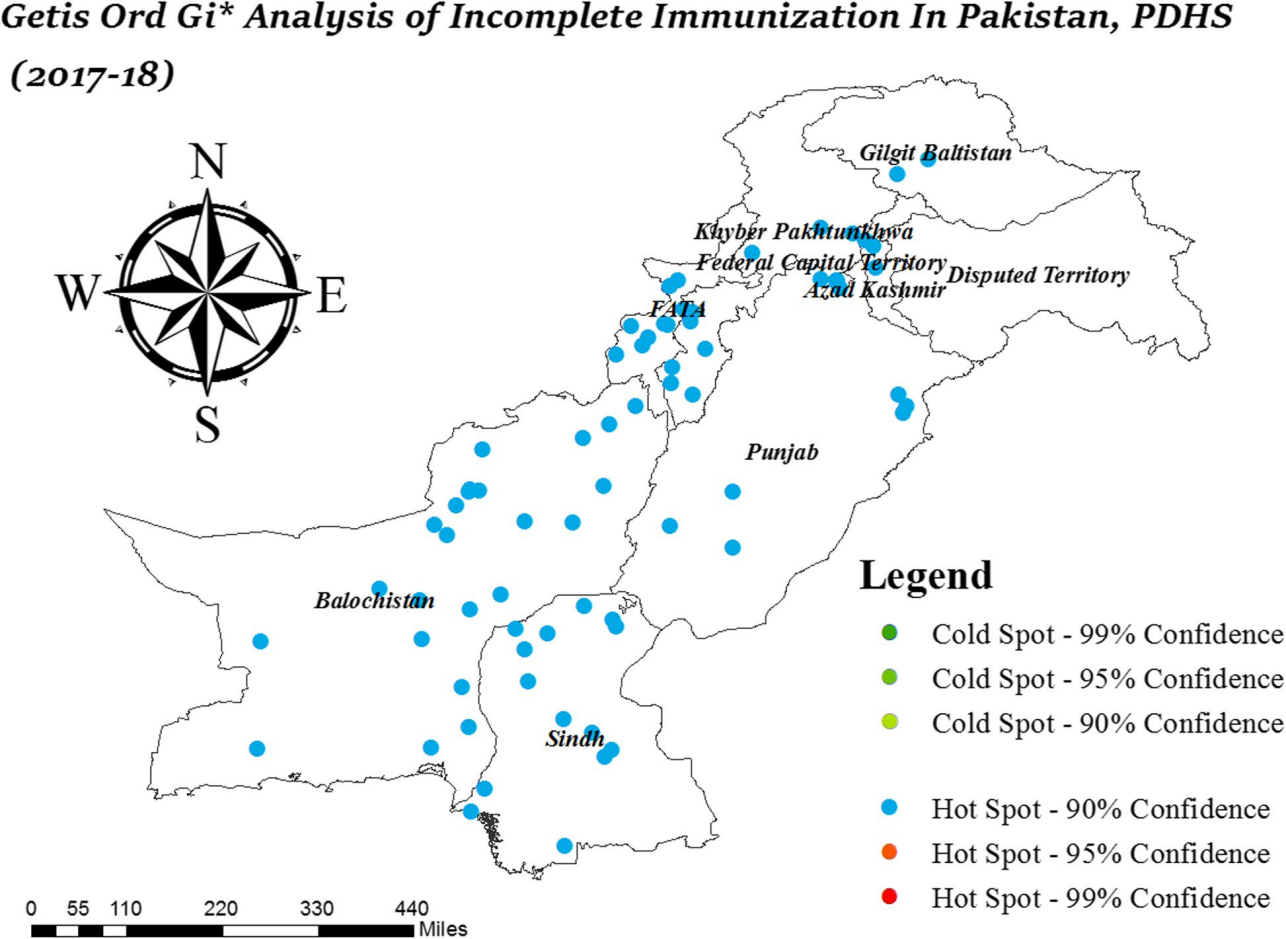
Hotspot Analysis of Incomplete Immunization in Pakistan; Shape file source: www.giszoo.com, Map output: own analysis on ArcGIS

The hotspot analysis detects the location of statistically significant spatial hot spot and cold spot clusters. It detects clusters with a high proportion of incomplete immunization as hot spots and clusters with a low proportion of incomplete immunization as cold spot. In Fig. 3, hotspot clusters of incomplete immunization were observed with only 90% confidence. In Balochistan, Sindh, East Punjab, South Punjab, Azad Jammu Kashmir, Federal Administered Tribal Areas (FATA), KPK and in Gilgit Baltistan, a high prevalence of incomplete immunization was observed. In Fig. 3, no cluster was observed with a low proportion of incomplete immunization.

### Spatial scan statistical analysis

The spatial scan statistical analysis has more power to identify hotspot clusters than other available hotspot analysis. The spatial scan statistical analysis identifies significant primary and secondary clusters of incomplete immunization. In Table 4, the first column represents primary clusters of incomplete immunization. These were the clusters with a high prevalence of incomplete immunization having longitude and latitude (35.458804 N, 74.763280 E) with a radius of 23.16 km in Gilgit Baltistan. The children who lived in these areas had 2.18 times more chances to become incompletely immunized.

**Table 4:** Spatial scan statistical analysis results of incomplete immunization in Pakistan, PDHS (2017–18)

Sr. no	Enumeration Blocks	Coordinate/radius	RR	LLR (p-value)
1	512, 507	(35.458804 N, 74.763280 E)/ 23.16 km	2.18	29.41* (0.0000)
2	335, 333, 331, 322, 327, 329, 323, 324, 326, 328, 330, 321, 332, 325, 359, 358, 355, 348, 339, 343, 337, 338, 347, 344, 336, 369, 340, 368, 356, 357, 353, 341, 352, 362, 351, 346, 361, 342, 350, 349, 411, 218, 364, 216, 365, 228, 229, 217, 412, 222, 226, 224, 406, 220, 83, 79, 219, 227, 408, 194, 371, 225, 413, 84, 192, 193, 230, 223, 410, 78, 237, 235, 231, 232, 233, 82, 200, 243, 239, 407, 195, 409, 234, 81, 238, 240, 80, 360, 314, 242, 201, 241, 198, 212, 202, 105, 313, 199 370, 75, 76	(30.802713 N, 66.526954 E)/ 457.19 km	1.57	21.20 * (0.0000)

In Gilgit Baltistan, a primary cluster of incomplete immunization was observed that had a high proportion of incomplete immunization. It is shown in Table 4 that the LLR of incomplete immunization was highest about at 29.41 for Gilgit-Baltistan. In Balochistan, South FATA, North Sindh, South KPK, and West Punjab, LLR was about 21.20. Children who lived in these areas had 1.57 times more chances to become incompletely immunized.

**Fig. 4:**
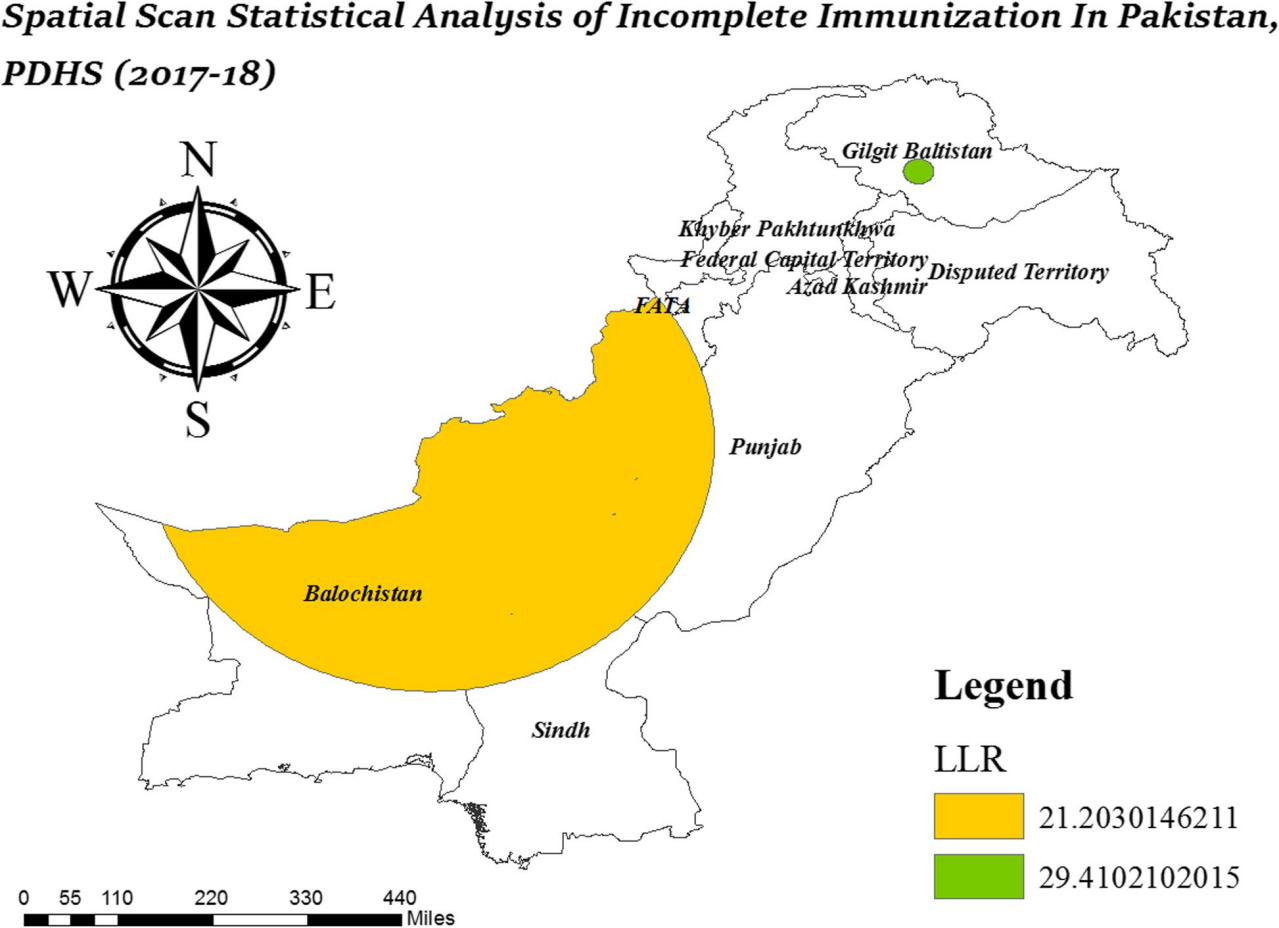
Spatial scan statistical analysis of incomplete immunization in Pakistan; Shape file source: www.giszoo.com, Map output: own analysis on ArcGIS

**Fig. 5:**
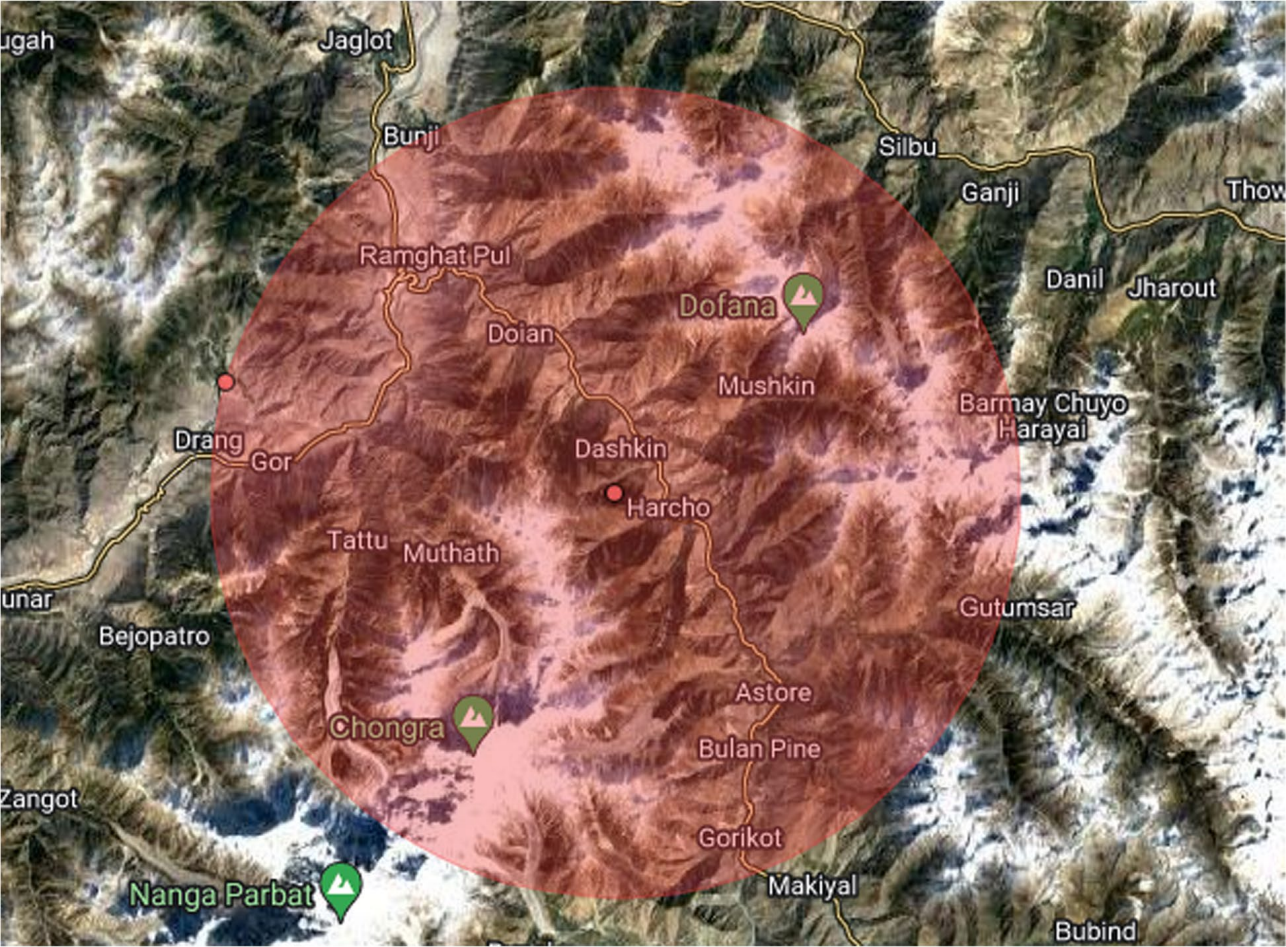
Primary clusters of incomplete immunization in Pakistan, PDHS (2017–18)

Figure 4 provides a cluster map which shows the area of significant clusters with a high risk of incomplete immunization. From Fig. 5, it can be seen that the significant hotspot areas with a high prevalence of incomplete immunization were identified in the area of Gilgit Baltistan, i.e., Harcho and Drang.

**Fig. 6:**
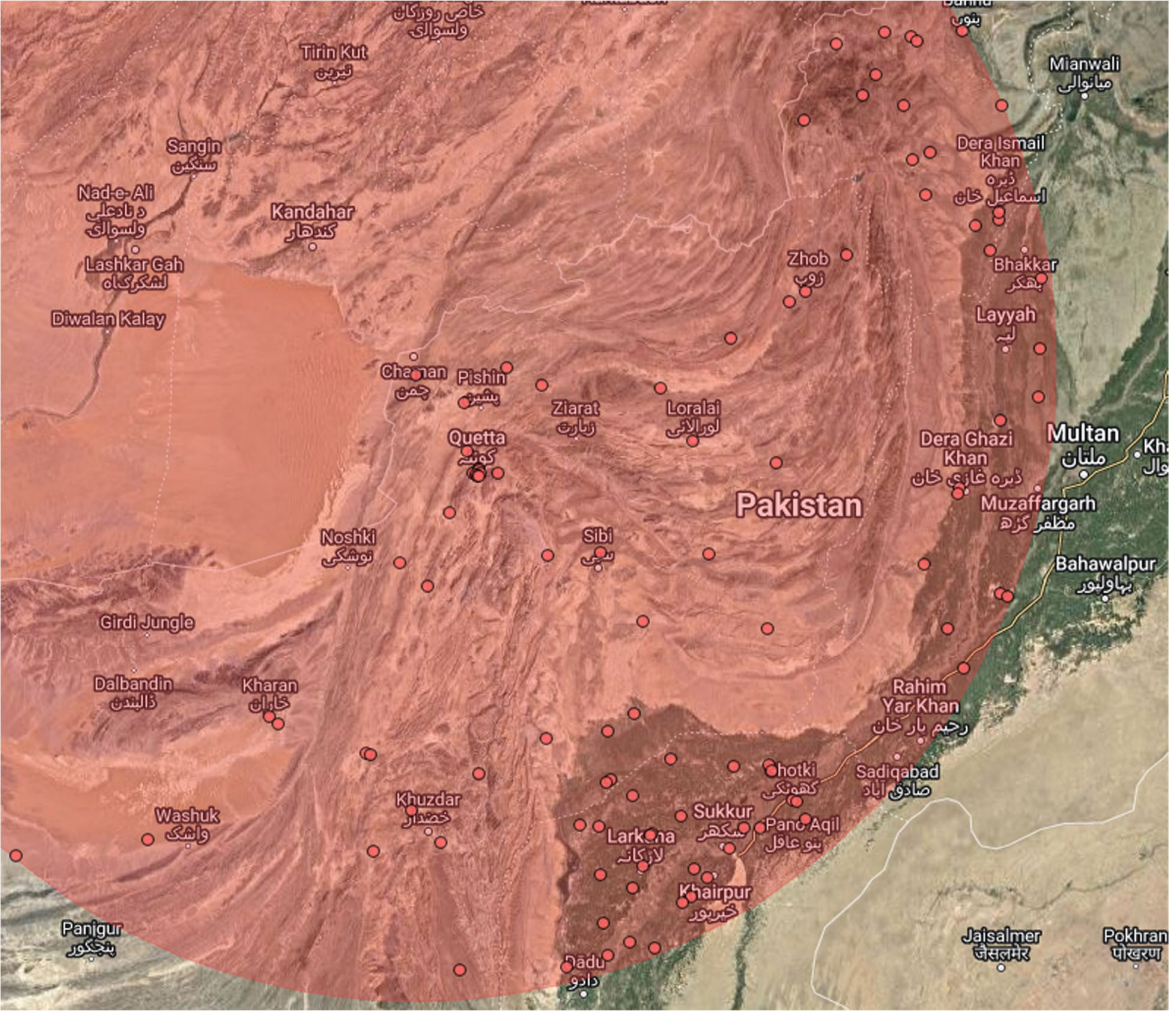
Secondary clusters of incomplete immunization in Pakistan, PDHS (2017–18)

In Fig. 6, secondary clusters of incomplete immunization are shown. The risk of incomplete immunization is low in secondary clusters as compared to primary clusters but it can’t be ignored. The children who resided in the second spatial window had 1.57 times more risk of incomplete immunization. The cities that were under the secondary spatial cluster are Quetta, Pishin, Kharan, Khuzdar, Larkana, Khairpur, Sukkar, Chaman, Loralai, Dadu, and DG Khan etc. (Fig. 6).

### Spatial interpolation

**Fig. 7:**
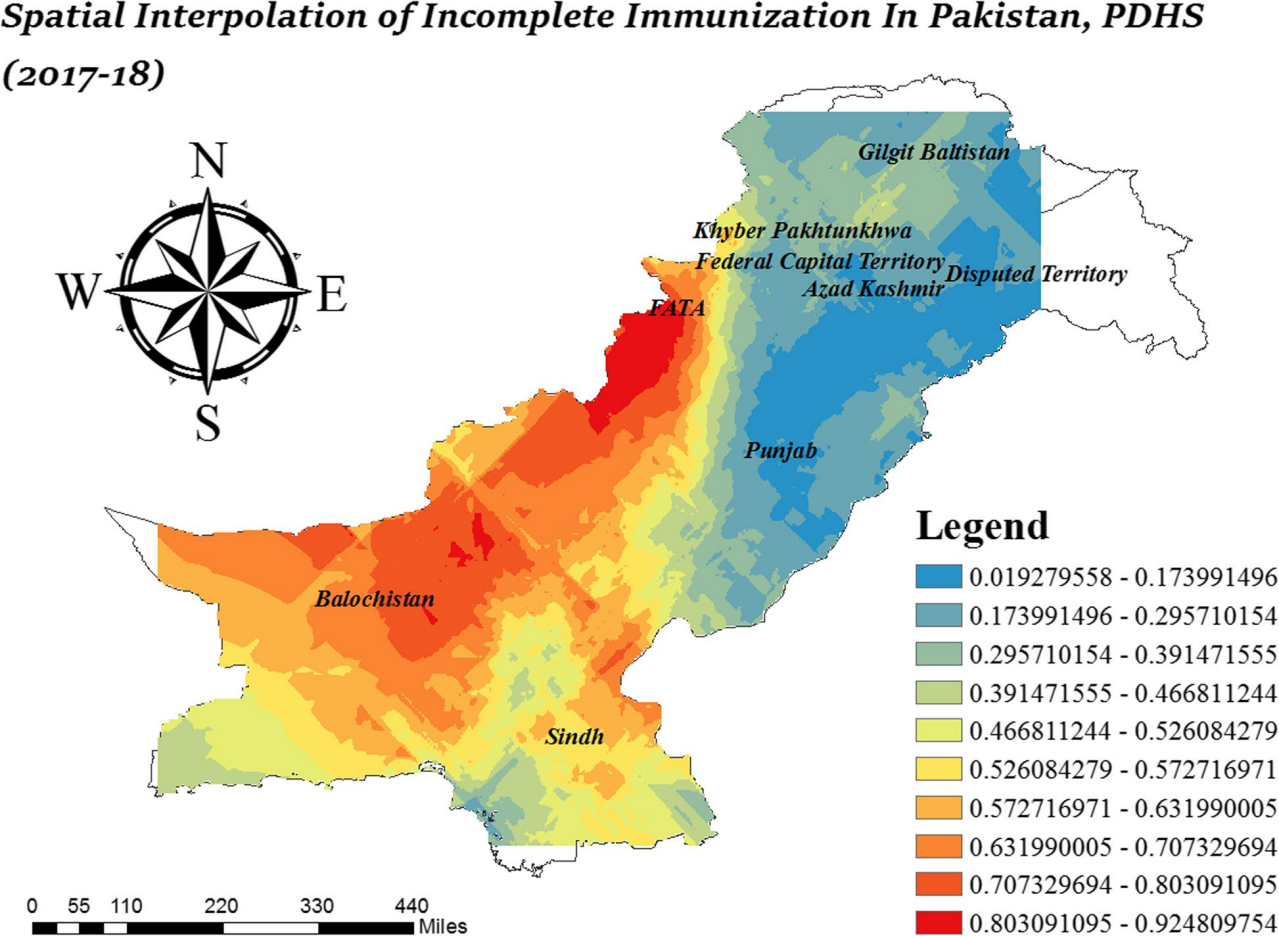
Ordinary kriging technique of interpolation for incomplete immunization in Pakistan; Shape file source: www.giszoo.com, Map output: own analysis on ArcGIS

Interpolation is a technique to determine the proportion of incomplete immunization in un-sampled areas on the basis of sampled areas. The ordinary Kriging technique was considered as best geostatistical interpolation technique and was selected to determine the distribution of un-sampled areas [24]. Figure 7, indicates the proportion of incomplete immunization varies from 80 to 92% in FATA. In central Balochistan, and around FATA the chance of incomplete immunization increased from 71 to 80%. Whereas the low risk areas were found in Punjab, Azad Jammu Kashmir, and East Gilgit Baltistan. In Sindh, the proportion of incomplete immunization varied from 47 to 70%.

### Geographically weighted regression

**Fig. 8:**
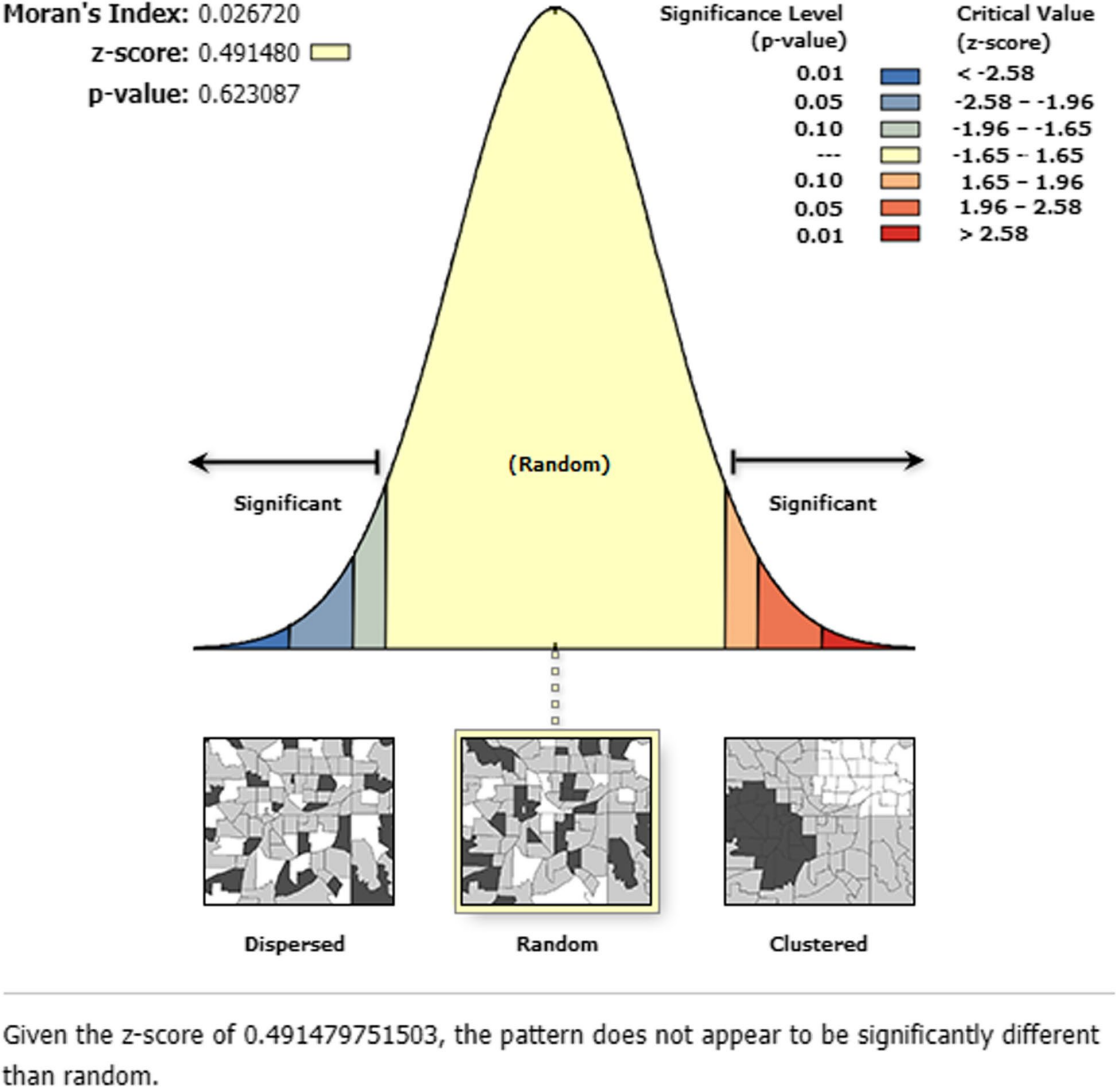
Spatially uncorrelated residuals of GWR, PDHS (2017–18)

In order to identify predictors of incomplete immunization and spatial behavior of these predictors, global geographically weighted regression along with local geographical weighted regression was performed. For GWR, firstly global geographical weighted regression was performed to check the assumptions and to identify the predictors. After that local geographical weighted regression was performed. Figure 8 describes spatially uncorrelated residuals of GWR.

### Global geographical weighted regression

Global geographical weighted regression helped to identify predictors of incomplete immunization as well as it helped to decide whether we were required to fit geographically weighted regression or not, based on assumptions. Before moving to global geographical weighted regression, exploratory regression was fitted. From exploratory regression, variables with 60% and above significance were selected for global geographical weighted regression. After checking the necessary assumptions of variables with p-value less than 0.05 were selected for local geographical weighted regression. Table 5 defines the results of Global geographical weighted regression coefficients for PDHS (2017–18). The global geographical weighted regression identifies female children, no media exposure, birth order >  = 7, and less than 4 antenatal visits as the predictors of incomplete immunization. A unit change in a female children, no media exposure, birth order >  = 7, and less than 4 antenatal visits changed the expected log odds of incomplete immunization by 0.107613, 0.305080, 0.550827, and 0.234893 respectively.

**Table 5:** Global Geographical weighted regression coefficients for PDHS (2017–18)

Variables	Coefficients	Probability	Robust Probability	VIF
**Intercept**	-0.049160	0.618869	0.777601	––-
**Female child**	0.107613	0.002473*	0.079447	2.03
**No Media Exposure**	0.305080	0.000000*	0.001091*	3.47
**Birth Order (> = 7)**	0.550827	0.000000*	0.015828*	1.84
**Antenatal Visits Less than 4**	0.234893	0.000000*	0.001628*	3.68

### Local geographical weighted regression

GWR software was used to fit geographically weighted regression. The GWR model was run to identify the accuracy of the model and to identify whether the geographical weighted regression model was better than the global geographical weighted regression or not. The AIC value computed for the geographically weighted regression model using the GWR software and it was 2158.05, which was lower than the AIC of global geographical weighted regression. The value of Adjusted R^2^ was also strong for the GWR model, given in Table 6, which shows that the GWR model was better than the global geographical weighted regression.

**Table 6:** Geographically weighted regression (Local Geographical Regression) diagnosis for PDHS (2017–18)

Diagnostic Criteria	Magnitude
**AICc**	2158.05
**R Squared**	0.71
**Adjusted R Squared**	0.70

**Fig. 9:**
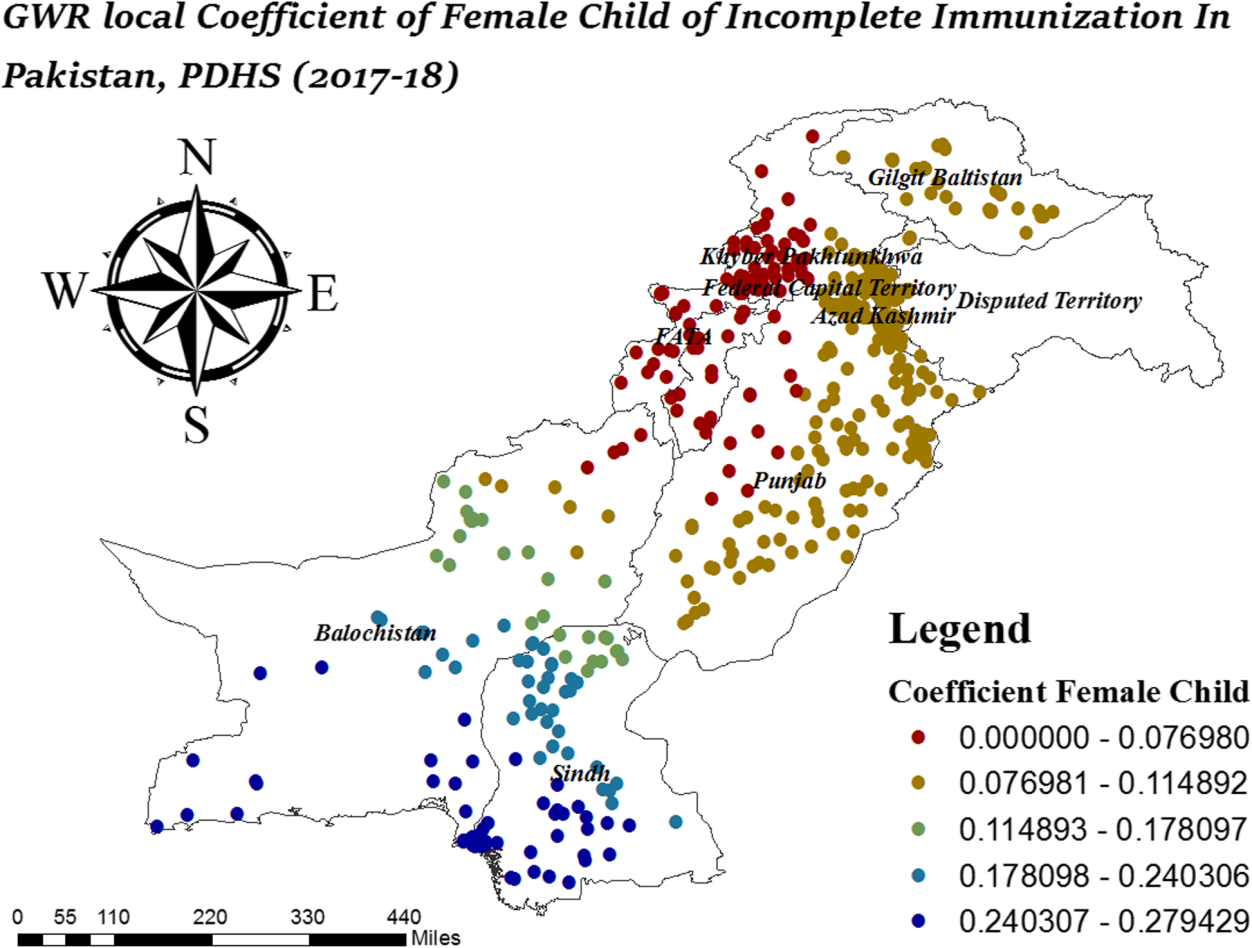
Local coefficient of female child for incomplete immunization in Pakistan, PDHS (2017–18); Shape file source: www.giszoo.com, Map output: own analysis on ArcGIS

**Fig. 10:**
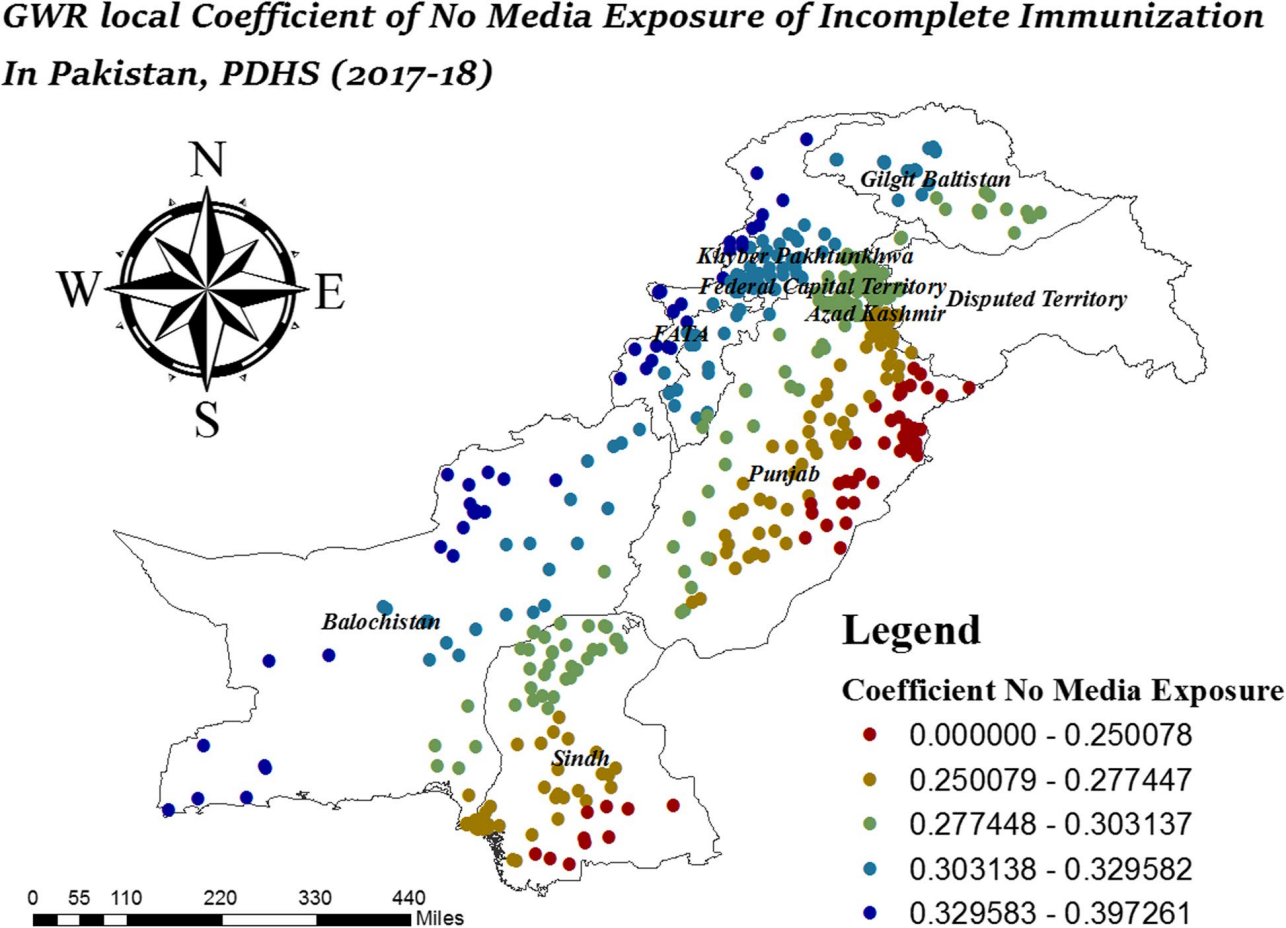
Local coefficient of no media exposure for incomplete immunization in Pakistan, PDHS (2017–18); Shape file source: www.giszoo.com, Map output: own analysis on ArcGIS

In Fig. 9, South Sindh and South Balochistan were the areas where female children had strong positive effects on incomplete immunization while female children had low effect on incomplete immunization in West Punjab, FATA, and KPK. In Fig. 10, FATA, North KPK, and Balochistan were the areas where no media exposure had a strong effect on incomplete immunization while the effect of no media exposure on incomplete immunization was low in East Punjab and South Sindh.

**Fig. 11:**
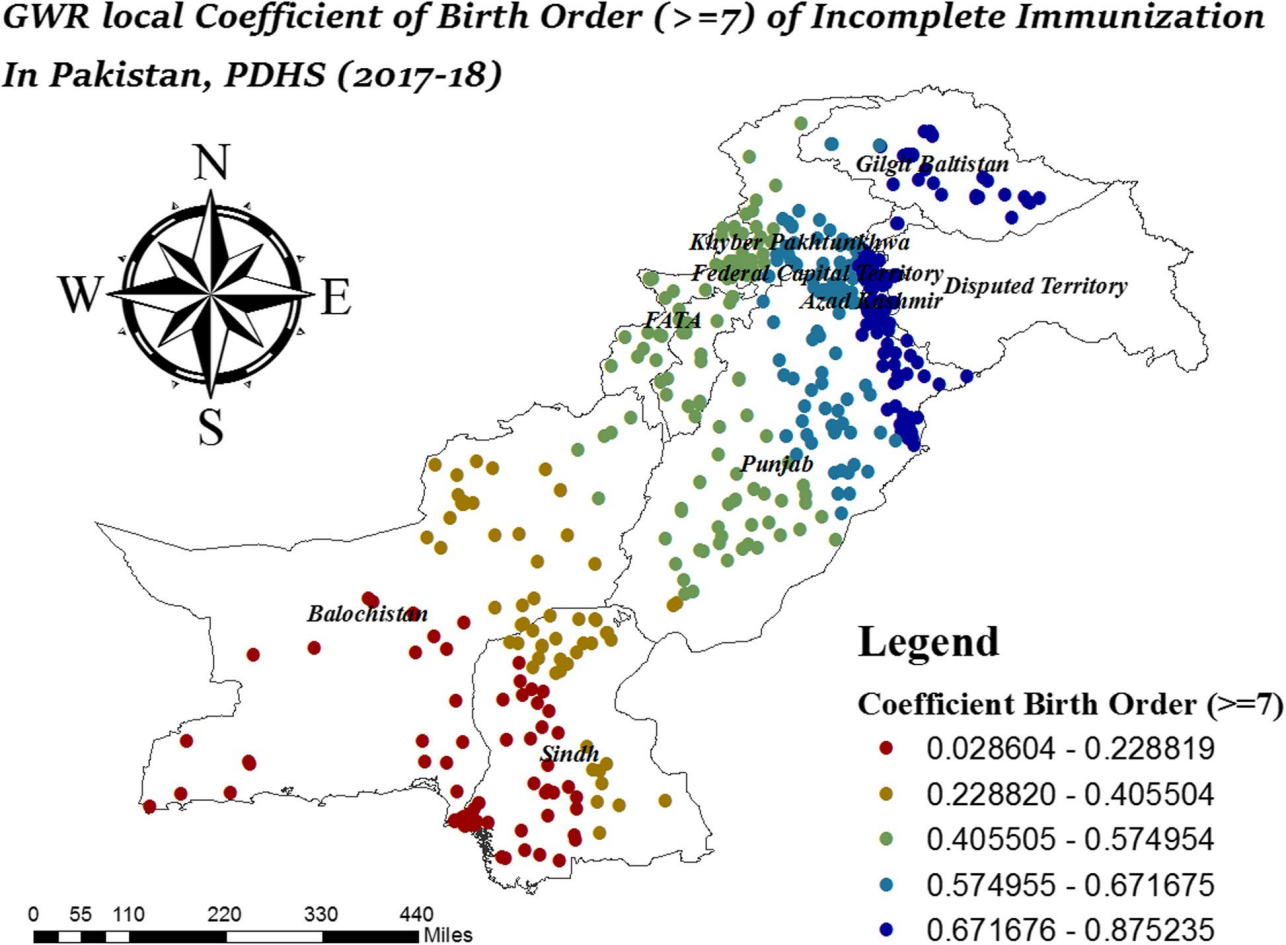
Local coefficient of birth order >  = 7 for incomplete immunization in Pakistan, PDHS (2017–18); Shape file source: www.giszoo.com, Map output: own analysis on ArcGIS

**Fig. 12:**
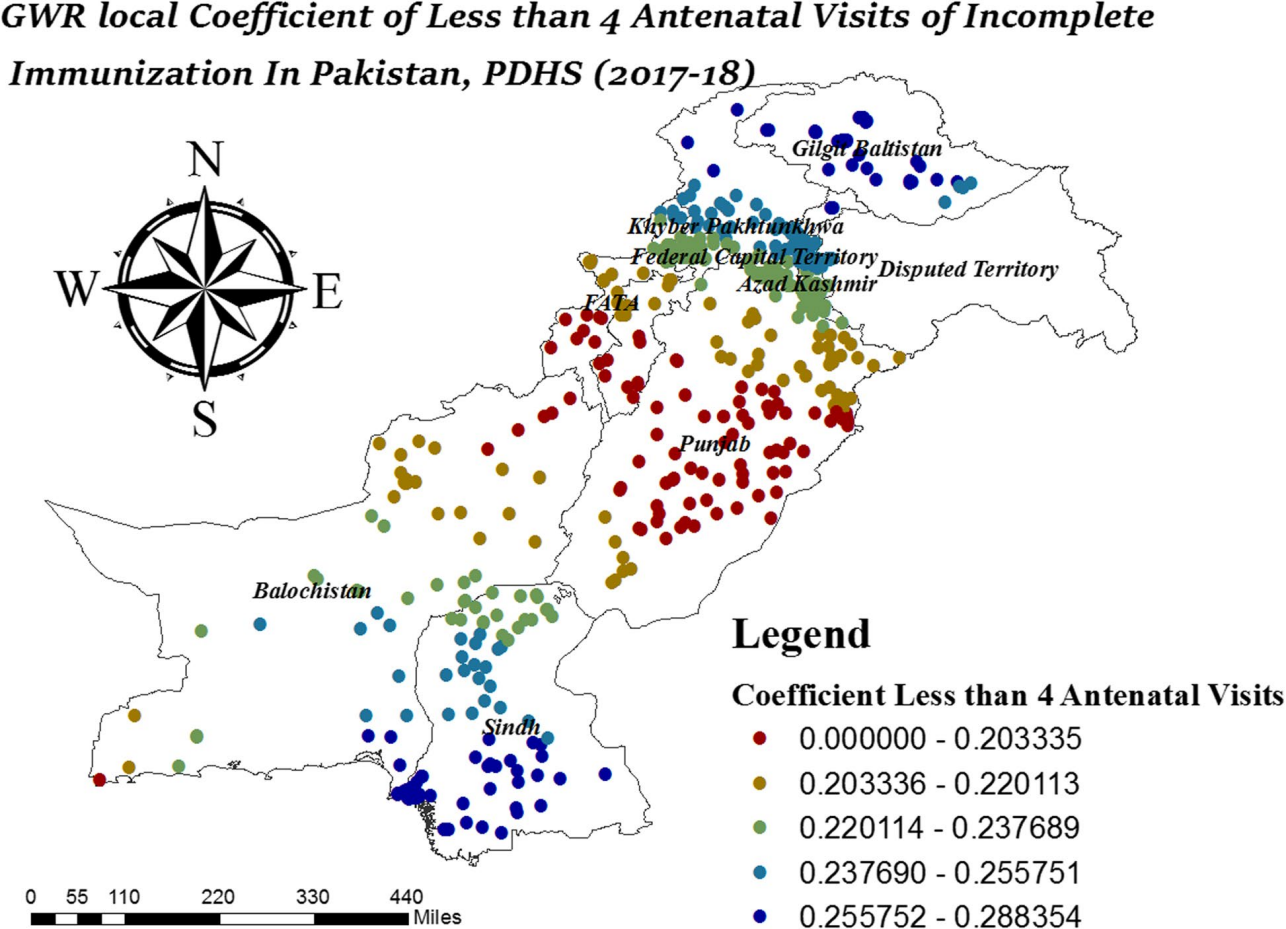
Local coefficient of less than 4 antenatal visits for incomplete immunization in Pakistan, PDHS (2017–18); Shape file source: www.giszoo.com, Map output: own analysis on ArcGIS

In Fig. 11, the variation in local coefficient birth order (> = 7) ranges from 0.028606 to 0.875235. It was observed, birth order (> = 7) had a strong coefficient of incomplete immunization in North Punjab, Gilgit Baltistan, and Azad Jammu Kashmir. In Fig. 12, incomplete immunization highly affected by less than four antenatal visits in Gilgit Baltistan, South Sindh, North KPK, South Balochistan and Azad Jammu Kashmir.

## Discussion

In spite of many efforts, incomplete immunization is still high in Pakistan. In the child nutrition and health sector, Pakistan’s progress has been substantially slower in comparison to all South Asian countries [26]. In Pakistan, the percentage of children born in health facilities increased substantially [27], and [1]. The effect of health facilities has an input on child immunization results that was the reason that 66% of children in Pakistan were vaccinated but still pace is low to achieve the target of 90% of vaccinated children till 2022.

A large number of female children were incompletely immunized in Pakistan [1]. Female children were at risk of incomplete immunization, the same results reported in many studies [28], and [29]. One of the major causes of incomplete immunization among female children was a prevalence of son preference in patriarchal societies. Due to gender biasedness attitude, female children remain deprived of basic health facilities as compared to male children. From the data of DHS India and other South Asian countries, it was found that the gender inequity has a significant effect on immunization coverage at all socio-economic levels [30]. Gender difference in access to health facilities is the major reason for avoidable deaths [30]. No association was found between mother’s occupation and incomplete immunization in this study similar to previous studies [29, 31]. It was found that working mothers had more chances to completely immunize their children because of high awareness and sensitization [32]. Educated, empowered, and employed mothers can fix up the trouble of incomplete immunization.

Mother’s education was repeatedly determined as the key indicator in child health and vaccination uptake [5, 28, 33]. children of uneducated parents were at more risk of incomplete immunization as compared to children of educated parents [34]. Low education lessens their ability to understand the importance of timely and complete immunization [33, 35–37]. Immunization is particularly linked with parents interaction with the health care system before the child birth [38, 39]. The antenatal visits before pregnancy have proven to be helpful for mothers to get information about child immunization [1]. This study reported that a mother who had less than four antenatal visits, the chances of incomplete immunization were high for her child [1]. It was noted that the child lived in a house where a female head had a low risk of incomplete immunization [14]. The reason may be that women by nature are observed to be more caring and more concerned about children health than men. The power of decision in the hand of the woman household head could be another reason.

Media exposure is the most compelling strategy for changing childhood vaccination behaviour. Media exposure identified as a significant predictor of incomplete immunization [40, 41]. Mothers who had media exposure have 41% less chances of incomplete immunization for their children. Similar results were found in other studies [27], and [42]. The child born to mothers aged 35 years and above were less likely to become incompletely immunized [43] as the women are quite mature and particular about immunization.

This study is in line with another study that also demonstrated significant regional variations regarding incomplete immunization in Pakistan [7]. Studies conducted in Uganda [44] and Africa [45] also reported regional differences regarding immunization of child. The risk of incomplete immunization was also found higher for children residing in rural areas as compared to urban areas in the current study and the same was observed in studies conducted in different regions of the world [46–48]. The reason is availability and accessibility of health facilities in urban areas [49].

The distribution of incomplete immunization was clustered spatially all over Pakistan. In some areas prevalence of incomplete immunization was high while the risk of incomplete immunization was also found quite low in certain areas. North Pakistan, and South West part of Pakistan was observed as a hotspot for incomplete immunization. In Sindh, Balochistan, South-West FATA and KPK, and Gilgit Baltistan, a high prevalence of incomplete immunization was observed. One reason is less expenditure spent on the health sector in Pakistan which was 20.9% of GDP in 2014–15 [50]. In Pakistan, at the provincial level, KPK and Balochistan spent the lowest portion of their public expenditure on the health sector [51, 52].

The primary cluster of incomplete immunization was found in Gilgit Baltistan. Spatial scan statistical analysis reported that children in Gilgit Baltistan were at higher risk of incomplete immunization as compared to other regions. In Gilgit Baltistan, access to health facilities is a major issue as people have to travel long for care facilities and specialized care [53]. In the last three decades road infrastructure has been improved in Gilgit Baltistan but still, people have to migrate to cities for education and good health facilities [54]. Drang and Harcho were the areas with a high prevalence of incomplete immunization in Gilgit Baltistan. The statistically significant secondary cluster spatial window was in Northern Balochistan and Sindh, East Punjab, South FATA and KPK.

Geographically weighted regression identified female child, no media exposure, birth order >  = 7, and less than 4 antenatal visits as the predictors of incomplete immunization. These predictors were contributing to the prevalence of incomplete immunization in Pakistan. In FATA and KPK, being a female child and having less than four antenatal visits has no higher effect on incomplete immunization as compared to other regions. The reason is that many organizations like USAID, UNICEF, UN Women, Aurat Foundation, PAIMAN and others are working on child and maternal health [1]. It was also found in the literature that less number of female child was unvaccinated in FATA as compared to male child [55]. It may also be due to the impact of programs, Government of Pakistan has started like the KPCSW program and gender program to empower women of West Pakistan [56].

### Conclusion and recommendations

The significant spatial heterogeneity of incomplete immunization was found across Pakistan. The spatial distribution of incomplete immunization was not random all over Pakistan. North Balochistan, North Sindh, South KPK and FATA, West Punjab, and Gilgit Baltistan were the significant hotspot areas for incomplete immunization. Drang and Harcho were identified as primary clusters of incomplete immunization in Gilgit Baltistan. Secondary cluster with a high risk of incomplete immunization were found in the region of Balochistan, Sindh and FATA.

Gender biasedness in the immunization of female children exists and its effect varied in different regions. Lack of media exposure had played a significant contribution towards incomplete immunization. Educated mothers and delivery of child at health facility had significantly reduced the risk of incomplete immunization.

Interventions like the provision of accessible public health facilities for the vaccination of children in hotspot areas should be the foremost step to increase the vaccine coverage. Government should also launch awareness programs with the partnership of media to educate people to discourage gender biasedness towards complete immunization of female children.

### Strength and limitations

The data were taken from PDHS 2017–18 and DHS datasets are considered as authentic data source in the area of public health. PDHS 2017–18 is the nationally representative survey. Software like ArcGIS, SaTScan, STATA, and GWR were used in this study. Findings and results obtained from these softwares complement and strengthen the objectives of the study to lead toward the final conclusion. Factors related to the environment, disease related and health system factors need to be created and assessed. These factors would help in for the better understanding of the reasons of incomplete immunization in Pakistan. In regard to spatial analysis, the SatScan analysis only identified the circular clusters, while irregular clusters were not detected.

## Data Availability

The data used for this study were publicly available (http://www.dhsprogram.com) with no personal identifier.
